# An Efficient Estimation Method for Reducing the Axial Intensity Drop in Circular Cone-Beam CT

**DOI:** 10.1155/2008/242841

**Published:** 2008-10-08

**Authors:** Lei Zhu, Jared Starman, Rebecca Fahrig

**Affiliations:** ^1^Department of Radiology, Stanford University, Stanford, CA 94305, USA; ^2^Department of Electrical Engineering, Stanford University, Stanford, CA 94305, USA

## Abstract

Reconstruction algorithms for circular cone-beam (CB) scans have been extensively studied in the literature. Since insufficient data are measured, an exact reconstruction is impossible for such a geometry. If the reconstruction algorithm assumes zeros for the missing data, such as the standard FDK algorithm, a major type of resulting CB artifacts is the intensity drop along the axial direction. Many algorithms have been proposed to improve image quality when faced with this problem of data missing; however, development of an effective and computationally efficient algorithm remains a major challenge. In this work, we propose a novel method for estimating the unmeasured data and reducing the intensity drop artifacts. Each CB projection is analyzed in the Radon space via Grangeat's first derivative. Assuming the CB projection is taken from a parallel beam geometry, we extract those data that reside in the unmeasured region of the Radon space. These data are then used as in a parallel beam geometry to calculate a correction term, which is added together with Hu's correction term to the FDK result to form a final reconstruction. More approximations are then made on the calculation of the additional term, and the final formula is implemented very efficiently. The algorithm performance is evaluated using computer simulations on analytical phantoms. The reconstruction comparison with results using other existing algorithms shows that the proposed algorithm achieves a superior performance on the reduction of axial intensity drop artifacts with a high computation efficiency.

## 1. INTRODUCTION

Circular cone-beam (CB) scans are commonly used in X-ray CT. However, given Tuy's data sufficiency condition [1], the Radon space data of the scanned object cannot be completely measured in such an imaging geometry, and an exact reconstruction is possible only in the plane of the source trajectory (midplane). Many approximate reconstruction algorithms have been proposed in the literature. The FDK algorithm, developed by Feldkamp et al. [2], is by far the most popular mainly due to its structure of one-dimensional (1D) shift-invariant filtering and backprojection. Although originally derived as a heuristic extension of the exact fan-beam reconstruction, the FDK algorithm has been shown to be equivalent to an exact 3D reconstruction if the unmeasured Radon space data are assumed zeros [3, 4], except that a small correction term is also needed [5, 6]. Therefore, the FDK algorithm is exact for an object with uniform distribution in the longitudinal direction [2], whose Radon space data (the first derivative of the Radon transform) are exactly zeros in the unmeasured region of a circular trajectory. However, in general, zero is not a good approximation of the missing Radon space data, for the case when the scanned object is nonuniform and compactly supported, and has nonnegative attenuation coefficients. Consequently, the reconstructed images have CB artifacts, such as the well-known intensity drop in the axial direction [2, 5, 7–10]. The reconstruction can be improved by using an auxiliary trajectory in addition to the circular trajectory to measure the missing data [11–13]. In this work, we focus on using circular trajectories only, and develop an estimation-based method to reduce the intensity drop artifacts.

Reduction of artifacts in circular cone-beam CT (CBCT) can be achieved by estimating the unmeasured data using interpolation or extrapolation. Using Grangeat's formula [3], CB projection data can be analyzed as the first derivative of the Radon transform of the scanned object, and data estimation using interpolation can be performed in the Radon space to suppress CB artifacts [8]. However, this method requires three-dimensional (3D) data gridding, and hence it is computationally intensive; and in addition the backprojection structure is lost. Estimation methods of the Radon space data are also proposed in the space of reconstructed images, using multiple scans with different source-to-axis distances [7, 14, 15]. Zeng et al. developed improved algorithms to reduce the intensity drop using iterations [16]. These algorithms require either multiple reconstructions using different imaging geometric parameters or an iterative reconstruction that involves several computationally intense forward and backprojection steps. Other researchers have developed many improved algorithms in the framework of filtered-backprojection (FBP), without using Grangeat's framework [9, 10, 17, 18]. Since FBP has a compact structure, it is not easy to implement data interpolation/extrapolation in the Radon domain to further reduce the intensity drop artifacts.

Hu discovered that in a circular CB trajectory, the original object can be written as the summation of three terms [5]:

(1) f = f^FDK + f^H + f^N,

where *f* is the original object, f^FDK is the FDK reconstruction, f^H is Hu's correction term which represents the information contained in a circular CB scan but not utilized in the FDK reconstruction, and ${\hat{f}}_{N}$ represents the information that is missing in the circular trajectory and cannot be reconstructed exactly. Hu proposed an algorithm that includes the first two measured terms, which shows reduced intensity drop in the reconstruction as compared to the FDK reconstruction [5]. Based on Grangeat's formula, Yang et al. proposed an algorithm that estimates the missing term ${\hat{f}}_{N}$ and effectively reduces the intensity drop artifacts [19]. However, the formula of the estimated term takes the form of shift-variant filtering and backprojection, two steps that both require intense computation.

The work presented in this paper is also based on Grangeat's formula and Hu's theory. However, the derivation of estimation formula for ${\hat{f}}_{N}$ is different from that of Yang, and the resulting implementation of the final formula is very efficient. We first analyze the CB projection data in the Radon domain via Grangeat's formula. Then, the unmeasured Radon space data are estimated from the CB projection by assuming that the projection is acquired in a parallel beam geometry. This approximation is equivalent to a data interpolation in the Radon domain. The estimated data are reconstructed as in the parallel beam geometry. More approximations are made to avoid the expensive steps of shift-variant filtering and backprojection in the calculation. The result is then added to the standard FDK reconstruction together with Hu's correction term to form the final reconstruction. Although the derivation assumes projections on a full scan, it can be readily extended to short-scan reconstructions using heuristic weighting schemes, such as Parker's weighting [20]. The performance of the proposed algorithm is verified using computer simulations on the 3D Shepp-Logan phantom, the FORBILD head phantom and the Defrise disc phantom. To fully evaluate the algorithm, the reconstructions are also compared with those using other existing algorithms.

The rest of this paper is organized as follows. Section 2 reviews the Radon transform and Grangeat's formula. The main algorithm is then derived and a reconstruction scheme is also proposed. Section 3 presents the results of computer simulations. Finally, Section 4 summarizes the paper.

## 2. METHOD

### 2.1. The system geometry

**Figure 1:**
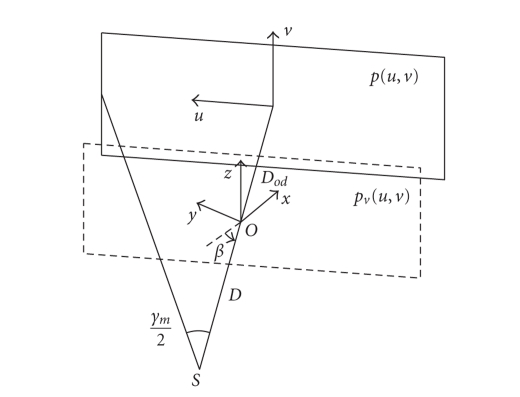
CB data acquisition geometry and coordinate system.

The system geometry is shown in Figure 1. In this paper, we use an equally spaced flat panel detector with a finite size. Algorithms when other types of detectors are used can be derived similarly. During data acquisition, the X-ray source *S* rotates about the *z* axis in the *x*-*y* plane, with a fixed distance *D* to the center of rotation *O*. Angle *γ*_*m*_ is the full fan angle determined by the size of the detector and the focal spot to detector distance. We derive the algorithm assuming that the range of the view angle *β* is 360 degrees in a full-scan mode. The detector is placed perpendicular to $\overset{\to }{SO}$ for each projection. The object to be reconstructed is described by a compactly supported nonnegative function $f(\vec{r})$, where $\vec{r}\;=\;(x,\;y,\;z)$ is the Cartesian coordinate. In the derivation, it is also assumed that there is no truncation of the projection data; this condition, however, will be relaxed based on the final formula.

Denoting the distance from *O* to detector as *D*_*od*_, the relationship between *p*(*u*, *v*, *β*), the real projection image, and *p*_*v*_(*u*, *v*, *β*), the image on a virtual detector that is parallel to the real detector and passes through *O*, is as follows:

(2) $$p_{v}(u,\;v,\;\beta )\;=\;p(u\frac{D\;+\;D_{od}}{D},\;v\frac{D\;+\;D_{od}}{D},\;\beta ).$$

For simplicity, we use *p*_*v*_ in the reconstruction hereafter, and the parameter *β* is dropped in the algorithm derivation if it is not used.

Main variables used in this paper are listed in Table 1 for clarity.

### 2.2. The Radon inversion and Grangeat's formula

Reconstruction of the original object from its projections can be solved using the 3D Radon inverse formula [3, 21]. However, it is not efficient to directly use the Radon inversion on the X-ray projection data. To reduce the computational complexity, Grangeat established a fundamental relationship between the X-ray projection image and the first derivative of the Radon transform of the scanned object.

**Figure 2:**
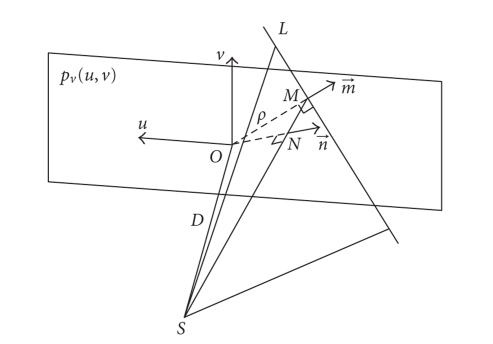
Illustration of the geometry of Grangeat's formula.

Figure 2 shows the geometric parameters of Grangeat's formula. One line *L* on the projection image *p*_*v*_ can be specified by two parameters, its distance to the origin $∥\;\overset{\to }{OM}\;∥\;=\;\rho$, and the vector $\vec{m}$ in the image plane and perpendicular to *L*. The line *L* and the focal spot *S* determine a plane *P*, which can also be specified by two parameters, its normal vector $\vec{n}$ and its displacement to the origin, $∥\;\overset{\to }{ON}\;∥\;=\;D\rho /\sqrt{D^{2}\;+\;\rho^{2}}$. Define an intermediate function *S* *p* as a weighted sinogram of the 2D projection image on the virtual detector *p*_*v*_:

(3) $$Sp(\rho ,\;\overset{\to }{m})\;=\;\int_{\overset{\to }{t}\in L(\rho ,\overset{\to }{m})}\frac{D}{\sqrt{D^{2}\;+\;u^{2}\;+\;v^{2}}}p_{v}(\overset{\to }{t})d\overset{\to }{t},$$

where $\vec{t}=\;(u,\;v)$ is a vector in the plane of the projection image, and $D/\sqrt{D^{2}\;+\;u^{2}\;+\;v^{2}}$ is the cosine weighting for an oblique incident angle.

Denote *R* *f* as the Radon transform of the scanned object *f*, which is specified by a unit vector ${\vec{n}}_{0}$ and a scalar *ρ*_0_:

(4) $$Rf(\rho_{0},\;{\overset{\to }{n}}_{0})\;=\;\int_{\overset{\to }{r}\in P_{0}(\rho_{0},{\overset{\to }{n}}_{0})}f(\overset{\to }{r})d\overset{\to }{r},$$

where *P*_0_ is a 2D plane, with a normal vector ${\vec{n}}_{0}$ and a displacement *ρ*_0_ from the origin.

The relationship between the first derivative of *S* *p* and the first derivative of *R* *f* can be found:

(5) $$\begin{array}{cc} & \frac{∥\overset{\to }{OS}{∥}^{2}}{∥\overset{\to }{NS}{∥}^{2}}S^{\prime }p(∥\overset{\to }{OM}∥,\;\overset{\to }{m})\;=\;R^{\prime }f(∥\overset{\to }{ON}∥,\;\overset{\to }{n})\\ & \Rightarrow \frac{\rho^{2}\;+\;D^{2}}{D^{2}}S^{\prime }p(\rho ,\;\overset{\to }{m})\;=\;R^{\prime }f(\frac{D\rho }{\sqrt{D^{2}\;+\;\rho^{2}}},\;\overset{\to }{n}), \end{array}$$

where both of the first derivatives are with regard to the first parameter.

**Figure 3:**
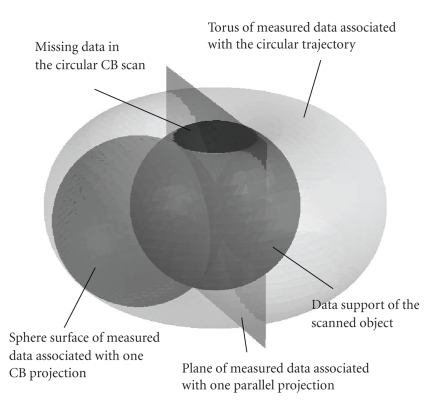
Data supports in the Radon domain.

Based on Grangeat's formula (5), Figure 3 shows the data supports in the domain of *R*′ *f* for a circular CB trajectory. For simplicity, hereafter we refer to the domain of the first derivative of the Radon transform as the Radon domain, and the data in this domain as the Radon space data. For one CB projection, the surface of a sphere is measured in the Radon domain; as the projection rotates, the sphere rotates as well, and after a full scan, a torus of data are measured. It can be seen that not all of the Radon space is measured, and the missing data problem is clear. The diameter of the sphere of one projection is *D*, the distance from the focal spot to the center of rotation. As this distance increases, the region of missing data gets smaller. In a parallel beam geometry (with an infinite *D*), this sphere surface becomes a plane, and the missing data problem disappears.

This formalism provides a clearer understanding of the three terms in (1), which is consistent with Hu's original arguments [5]. The FDK reconstruction f^FDK represents all the information inside the torus of measured data and partial information on the surface of the torus; Hu's correction term ${\hat{f}}_{H}$ compensates for the unused data on the surface of the torus; the term ${\hat{f}}_{N}$ represents the missing information outside the torus, and it is our goal to estimate these data.

### 2.3. The algorithm derivation

The missing data represented by the term ${\hat{f}}_{N}$ result in CB artifacts in the reconstructed images. One solution to this problem is estimating this term using interpolation in the Radon domain. Inspired by the fact that a parallel beam geometry is free of the missing data problem, the missing data are estimated from measured CB projections, assuming that they are acquired in a parallel geometry. Then reconstruction is carried out using only these estimated data as though acquired in a parallel beam geometry, and the result is added to the standard FDK term and Hu's term as a correction to form a final reconstruction. Using this method, partial data on the spherical surface of one CB projection as shown in Figure 3 are filled in the region of missing data, and a data interpolation/extrapolation is carried out implicitly.

**Figure 4:**
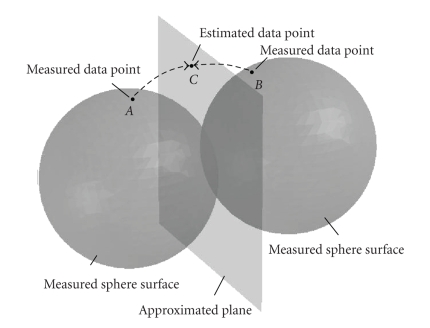
Illustration of the concept of implicit data interpolation/extrapolation. Data point *C* on the approximated plane is a weighted summation of measured data points *A* and *B*.

The concept of implicit data interpolation/extrapolation is illustrated in the Radon space in Figure 4. As similar to Figure 3, the sphere surfaces in the figure are measured using CB projections. If we assume the CB projections are acquired in a parallel beam geometry and reconstruct the object using a parallel beam geometry as well, the measured sphere surfaces become planes, that is, data points on the measured sphere surfaces are relocated onto the planes. Note that measured sphere surfaces associated with CB projections from the opposite projection angles are approximated as the same plane in the Radon space. As shown in Figure 4, data point *C* on the plane maps to two points *A* and *B* associated with two different CB projections. After the CB to parallel approximation, both data points *A* and *B* are “moved” to data point *C*; equivalently, the approximated data point *C* is a weighted summation of data points *A* and *B*. Therefore, this process can be considered as a linear interpolation of data point *C* from measured data points *A* and *B*.

**Figure 5:**
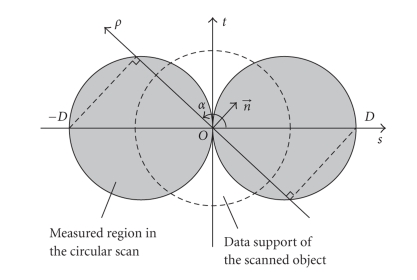
A vertical plane in the Radon domain.

Using the idea of implicit interpolation/extrapolation, we can estimate Radon space data on a plane from two CB projections. As the projection angle changes, all the Radon space data can be estimated. However, we only need to estimate the data in the missing region of the Radon space in a CB geometry, and use them in the correction of the artifacts. Figure 5 is a 2D plot of the vertical plane in the Radon domain in Figure 3. If a parallel beam geometry is used in the data acquisition, the data of this plane can be measured completely using one projection from the direction of the normal of the plane. In a CB geometry, however, the measured data in the plane consists of two discs (shown as shaded regions), and the data outside are missing. If the CB projection from the direction of the normal of the plane is assumed to be a parallel projection, the measured data can be calculated in the whole plane; then only the data outside the shaded region are used in the reconstruction of the correction term ${\hat{f}}_{N}$. The data of *R*′ *f* in the missing region can be separated from the measured data by multiplying by *w*(*s*, *t*), a map function that is zero in the shaded region of Figure 5, and one outside. Denote *p*_*p*_ as the projection image in a parallel beam geometry from the direction of the normal of the plane, and *S* *p*_*p*_ as its sinogram. The relationship between *R*′ *f* and *S*′ *p*_*p*_ can be found using Grangeat's formula, by letting *D* go to infinity in (5):

(6) $$S^{\prime }p_{p}(\rho ,\;\overset{\to }{m})\;=\;R^{\prime }f(\rho ,\;\overset{\to }{n}).$$

From Figure 2, it is also seen that the unit vector $\vec{m}$ becomes identical to $\vec{n}$ in a parallel geometry. Let $\vec{m}$ or $\vec{n}=\;(\cos\alpha ,\;\sin\alpha )$. For simplicity, the functions $S^{\prime }p_{p}(\rho ,\;\vec{m})$ and $R^{\prime }f(\rho ,\;\vec{n})$ are rewritten as *S*′ *p*_*p*_(*ρ*, *α*) and *R*′ *f*(*ρ*, *α*) in the polar coordinate system. Based on Figure 5, the map function *w* in the (*ρ*, *α*) coordinate system can be found as

(7) $$w(\rho ,\alpha )\;=\;\{\begin{array}{ll} 1, & |\;\rho \;|\;>\;|D\cos\alpha |,\\ 0, & \text{otherwise}. \end{array}$$

Now the multiplication of *w*(*ρ*, *α*) on *R*′ *f*(*ρ*, *α*) can be done directly on *S*′ *p*_*p*_, according to (6):

(8) $$S^{\prime }p_{p}(\rho ,\;\alpha )w(\rho ,\;\alpha )\;=\;R^{\prime }f(\rho ,\;\alpha )w(\rho ,\;\alpha ).$$

We then approximate the parallel projection data *S*′ *p*_*p*_ using the CB projection data *S*′ *p*. Mathematically, the following approximation is made:

(9) $$\frac{\rho^{2}\;+\;D^{2}}{D^{2}}S^{\prime }p(\rho ,\;\alpha )w(\rho ,\;\alpha )\;\approx \;S^{\prime }p_{p}(\rho ,\;\alpha )w(\rho ,\;\alpha ).$$

Note that *D* goes to infinity in a parallel beam geometry, and the weight (*ρ* ^2^ + *D* ^2^)/*D* ^2^ equals one on the right-hand side. Equations (8) and (9) show that the missing Radon space data are approximated using the measured CB projection data.

We first derive the correction term that compensates for the missing data in terms of *S*′ *p*_*p*_, and then apply the approximation shown in (9). To calculate the correction term, reconstruction must be carried out using only partial projection data in the domain of *S*′ *p*_*p*_. This can be done by using a 2D reconstruction of the projection data, followed by a 3D parallel reconstruction. Note that these intermediate steps are used only in the derivation, and the derived final formula is implemented efficiently without these steps.

Since *S* *p*_*p*_ is the sinogram of the projection image, we can compute the parallel projection image *p*_*p*_ using FBP in a 2D parallel geometry:

(10) $$\begin{array}{ll} p_{p}(u,\;v) & \;=\;\frac{1}{2}\int_{0}^{2\pi }\int_{-\infty }^{\infty }Sp_{p}(\rho ,\;\alpha )g_{0}(u\cos\alpha \;+\;v\;\sin\;\alpha \;-\;\rho )d\rho \;d\alpha \end{array}$$

(11) $$\begin{array}{ll} & =\;\frac{1}{4\pi }\int_{0}^{2\pi }\int_{-\infty }^{\infty }S^{\prime }p_{p}(\rho ,\;\alpha )g_{h}(u\cos\alpha +v\;\sin\;\alpha \;-\;\rho )d\rho \;d\alpha , \end{array}$$

where *g*_0_(*u*) is the ramp-filter kernel and *g*_*h*_(*u*) = 1/*π* *u* is the Hilbert kernel. In (11), we use a Hilbert transform after a first derivative operation to substitute for the ramp-filtering, so that the calculation can be directly applied on *S*′ *p*_*p*_.

Denote *p*_*m*_ as the projection image reconstructed from partial data of *S*′ *p*_*p*_. Using (11), we have

(12) $$p_{m}(u,v)=\frac{1}{4\pi }\int_{0}^{2\pi }\int_{-\infty }^{\infty }S^{\prime }p_{p}(\rho ,\alpha )w(\rho ,\alpha )\times g_{h}(u\cos\alpha +v\;\sin\;\alpha -\rho )d\rho \;d\alpha$$

Denote ${\hat{f}}_{c}$ as an estimate of the missing data ${\hat{f}}_{N}$ in (1). We can compute the correction term ${\hat{f}}_{c}$ using the 3D parallel reconstruction of *p*_*m*_. This computation can be done as slices of 2D parallel reconstructions. The FBP-based reconstruction is

(13) f^c(x, y, z) = 12∫02πpF(xcosβ + y sin β, z, β)dβ,

where *p*_*F*_ is the ramp-filtered parallel projection at view angle *β*:

(14) $$\begin{array}{ll} p_{F}(u,\;v) & \;=\;\int_{-\infty }^{\infty }p_{m}(\overset{¯}{u},\;v)g_{0}(u\;-\;\overset{¯}{u},\;v)d\overset{¯}{u} \end{array}$$

(15) $$\begin{array}{ll} & =\;\frac{1}{2\pi }\int_{-\infty }^{\infty }p_{m}^{\prime }(\overset{¯}{u},\;v)g_{h}(u\;-\;\overset{¯}{u},\;v)d\overset{¯}{u}. \end{array}$$

Calculation of ${\hat{f}}_{c}$ using (12), (13), and (15) can be simplified. Based on the two-step method developed by Noo et al. [22], 2D parallel reconstruction can be carried out using a derivative backprojection followed by a 1D Hilbert transform on the reconstructed image. Therefore, the Hilbert transform is taken out of the integral:

(16) $$\begin{array}{ll} & p_{m}(u,\;v)\\ & \;\;=\;\frac{1}{4\pi }\int_{-\infty }^{\infty }g_{h}(\overset{¯}{u})\int_{0}^{2\pi }\text{sgn}(\cos\alpha )\\ & \;\;\;\;\;\;\;\;\;\;\;\;\times {S^{\prime }\;p}_{p}((u\;-\;\overset{¯}{u})\cos\alpha \;+\;v\;\sin\;\alpha ,\;\alpha )\\ & \;\;\;\;\;\;\;\;\;\;\;\;\times w((u-\overset{¯}{u})\cos\alpha +v\;\sin\;\alpha ,\;\alpha )d\alpha \;d\overset{¯}{u}, \end{array}$$

where sgn is the signum function.

Define the function *p*_*h*_ as

(17) $$p_{h}(u,\;v)\;=\;\int_{0}^{2\pi }\text{sgn}(\cos\alpha )S^{\prime }p_{p}(u\cos\alpha \;+\;v\;\sin\;\alpha ,\alpha )\;\times \;w(u\cos\alpha \;+\;v\;\sin\;\alpha ,\alpha )d\alpha .$$

Then, we have

(18) $$p_{m}^{\prime }(u,\;v)\;=\;\frac{1}{4\pi }\int_{-\infty }^{\infty }g_{h}(\overset{¯}{u})p_{h}^{\prime }(u\;-\;\overset{¯}{u},\;v)d\overset{¯}{u}.$$

Both first derivatives are with regard to the first parameter.

Insert (18) into (15), and the two Hilbert transforms become −1 since the Hilbert transform of a Hilbert transform of a function equals the negative of the function:

(19) $$p_{F}(u,\;v)\;=\;-\frac{1}{8\pi^{2}}p_{h}^{\prime }(u,\;v).$$

The problem is simplified to the calculation of *p*_*h*_′. Applying the approximation in (9), we have

(20) $$\begin{array}{ll} & p_{h}^{\prime }(u,v)\\ & \approx \frac{\partial }{\partial u}(\int_{0}^{2\pi }\text{sgn}(\cos\alpha )\frac{{\left(u\cos\alpha +v\;\sin\;\alpha \right)}^{2}+D^{2}}{D^{2}}\\ & \;\;\;\;\;\times S^{\prime }p(u\cos\alpha +v\;\sin\;\alpha ,\alpha )w(u\cos\alpha +v\;\sin\;\alpha ,\alpha )d\alpha )\\ & =\int_{0}^{2\pi }\text{sgn}(\cos\alpha )\frac{{\left(u\cos\alpha +v\;\sin\;\alpha \right)}^{2}+D^{2}}{D^{2}}\\ & \;\;\;\;\;\times \frac{\partial }{\partial u}(S^{\prime }p(u\cos\alpha +v\;\sin\;\alpha ,\alpha ))w(u\cos\alpha +v\;\sin\;\alpha ,\alpha )d\alpha \\ & \;+\int_{0}^{2\pi }\text{sgn}(\cos\alpha )\frac{\partial }{\partial u}(\frac{{\left(u\cos\alpha +v\;\sin\;\alpha \right)}^{2}+D^{2}}{D^{2}})\\ & \;\;\;\;\;\times S^{\prime }p(u\cos\alpha +v\;\sin\;\alpha ,\alpha )w(u\cos\alpha +v\;\sin\;\alpha ,\alpha )d\alpha \end{array}$$

(21) $$\begin{array}{ll} & =\int_{0}^{2\pi }\text{sgn}(\cos\alpha )\frac{{\left(u\cos\alpha +v\;\sin\;\alpha \right)}^{2}+D^{2}}{D^{2}}\cos\alpha \\ & \;\;\;\;\;\times S^{\prime \prime }p(u\cos\alpha +v\;\sin\;\alpha ,\alpha )w(u\cos\alpha +v\;\sin\;\alpha ,\alpha )d\alpha \\ & \;+\int_{0}^{2\pi }\text{sgn}(\cos\alpha )\frac{2(u\cos\alpha +v\;\sin\;\alpha )}{D^{2}}\cos\alpha \\ & \;\;\;\;\;\times S^{\prime }p(u\cos\alpha +v\;\sin\;\alpha ,\alpha )w(u\cos\alpha +v\;\sin\;\alpha ,\alpha )d\alpha \\ & =\int_{0}^{2\pi }|\cos\alpha |w(u\cos\alpha +v\;\sin\;\alpha ,\alpha )\\ & \;\;\;\;\;\times (\frac{{\left(u\cos\alpha +v\;\sin\;\alpha \right)}^{2}+D^{2}}{D^{2}}S^{\prime \prime }p(u\cos\alpha +v\;\sin\;\alpha ,\alpha )\\ & \;\;\;\;\;+\frac{2(u\cos\alpha +v\;\sin\;\alpha )}{D^{2}}S^{\prime }p(u\cos\alpha +v\;\sin\;\alpha ,\alpha ))d\alpha . \end{array}$$

The discontinuous positions of the function *w* correspond to the points on the surface of the torus of measured data in Figure 3, which are compensated for by Hu's correction term. Therefore, in (20), the derivatives on the discontinuities of *w* are not included.

Equation (21) has a structure of shift-variant filtering (due to the shift-variance of the multplication by the weighting function) and backprojection, therefore the implementation is not very efficient. Furthermore, since a weighted sinogram of each 2D projection image is needed in the calculation, it is required that no truncation is present in the projection. In particular, the method suffers from projection truncation in the longitudinal direction, so-called long object problem.

We simplify (21) using further approximations. Since the weighting of *S*′′ *p* is usually much larger than that of *S*′ *p*, that is, (*ρ* ^2^ + *D* ^2^)/*D* ^2^ versus 2*ρ*/*D* ^2^, the second term associated with *S*′ *p* is ignored. For a circular trajectory with a not very large cone angle, we have *D* ≫ *ρ*. The weighting function *w* is nonzero only when |cos*α*| < |*ρ*/*D*| , that is, in small neighborhoods of *α* at *π*/2 and 3*π*/2. In these neighborhoods, the following approximations can be made:

(22) $$\begin{array}{c} v\;\sin\;\alpha \approx \text{sgn}(\sin\;\alpha )v,\\ u\cos\alpha \approx 0,\\ S^{\prime \prime }p(\text{sgn}(\sin\;\alpha )v,\alpha )\approx S^{\prime \prime }p(v,\frac{\pi }{2}). \end{array}$$

Now the calculation of *p*_*h*_′ can be simplified as

(23) $$\begin{array}{ll} p_{h}^{\prime }(u,\;v) & \approx 2\int_{0}^{\pi }|\cos\alpha |w(v,\;\alpha )\frac{v^{2}\;+\;D^{2}}{D^{2}}S^{\prime \prime }p(v,\;\alpha )d\alpha \\ & \approx 2\frac{v^{2}\;+\;D^{2}}{D^{2}}S^{\prime \prime }p(v,\;\frac{\pi }{2})\int_{\pi /2-\arccos(|v|/D)}^{\pi /2+\arccos(|v|/D)}|\cos\alpha |d\alpha \\ & =\;4\frac{v^{2}\;+\;D^{2}}{D^{2}}(1\;-\;\frac{\sqrt{D^{2}-v^{2}}}{D})S^{\prime \prime }p(v,\;\frac{\pi }{2})\\ & =\;4\frac{v^{2}+\;D^{2}}{D^{2}}(1\;-\;\frac{\sqrt{D^{2}-v^{2}}}{D})\\ & \;\times \;\frac{\partial^{2}}{\partial v^{2}}(\int_{-\infty }^{\infty }\frac{D}{\sqrt{{\overset{¯}{u}}^{2}+v^{2}+D^{2}}}p_{v}(\overset{¯}{u},v,\beta )d\overset{¯}{u}). \end{array}$$

The correction term ${\hat{f}}_{c}$ can be calculated by combining (13), (19), and (23).

Note that the approximate *p*_*h*_′ (or *p*_*F*_) is a function independent of parameter *u*. Backprojection of *p*_*F*_ shown in (13) can be implemented efficiently by simply changing variable *v* in the projection space to *z* in the reconstruction space.

### 2.4. Practical reconstruction scheme

The final reconstruction is the summation of the FDK reconstruction f^FDK, Hu's term ${\hat{f}}_{H}$, and the correction term ${\hat{f}}_{c}$:

(24) f^ = f^FDK + f^H + f^c.

The practical implementation of this formula is summarized below. The derivations of the FDK reconstruction and Hu's term can be found in [2, 5], respectively, and the formulae are presented here as a reference:

(25) f^FDK(x,y,z)=12∫02π(Dx+D)β2qF((Dyx+D)β,(Dzx+D)β,β)dβ,qF(u,v,β)=∫−∞∞Du¯2+v2+D2pv(u¯,v,β)g0(u−u¯)du¯,

where the subscript *β* stands for the coordinate transformation of rotation about the *z* axis by *β*; and

(26) f^H(x,y,z)=−12π∫02π(z(x+D)2)βqD((Dzx+D)β,β)dβ,qD(v,β)=12π∂∂v∫−∞∞Du¯2+v2+D2pv(u¯,v,β)du¯.

${\hat{f}}_{c}$ is calculated by using (13), (19), and (23):

(27) f^c(x,y,z)=−14π2z2+D2D2(1−D2−z2D) ×∫02π∂2∂z2(∫−∞∞Du¯2+z2+D2pv(u¯,z,β)du¯)dβ.

This estimation formula of the missing term ${\hat{f}}_{N}$ in (1) is the main result of the paper. The equation shows a simple structure of calculation. Note that, since the second derivative operation is very sensitive to high-frequency errors and the intensity drop artifacts are mostly low-frequency signals in the longitudinal direction, filtering techniques are used to suppress the errors in the calculation. A practical implementation can be divided into the following steps.

**Step 1.**

Take each projection *p*_*v*_(*u*, *v*, *β*), integrate along the direction of *u*.

**Step 2.**

Take the second derivative with respect to variable *v*.

**Step 3.**

Filter the 1D profile obtained from Step 2 using a median filter and a window filter.

**Step 4.**

Integrate the processed 1D profile along the projection angle *β*.

**Step 5.**

Change coordinate variable from the projection space to the reconstruction space (from *v* to *z*).

**Step 6.**

Weight by $\left(-1/4\pi^{2})((z^{2}\;+\;D^{2})/D^{2})(1\;-\;\sqrt{D^{2}\;-\;z^{2}}/D\right)$.

**Step 7.**

Replicate the 1D profile of *z* in the directions of *x* and *y* to generate a 3D volume.

As discussed earlier, Step 3 is important to suppress high-frequency misestimation and remove the streak artifacts that are otherwise present in ${\hat{f}}_{c}$. The median filter is able to remove high spikes caused by object boundaries, and the window filter is able to smooth out small fluctuations. In all the implementations presented in this paper, we used a median filter with a width of 10 pixels and a Hamming window filter. It is worth mentioning that since the filtering is applied only on ${\hat{f}}_{c}$ to enforce low-frequency estimation, it will not affect the resolution of the reconstructed images obtained by the first two terms in (24).

In (27), we take the second derivative of the projection images along the vertical direction, and therefore the proposed algorithm can survive the long object problem. The calculation of (27) is also very efficient, since neither a shift-variant filtering step nor a backprojection step is used. This feature makes the proposed algorithm distinct from other existing algorithms, such as Yang's method [19]. As will be shown in the section of numerical results, in our implementations, the proposed method is typically 7-8 times more efficient than Yang's method. Note that the calculation of Hu's term (26) has the same FBP structure as the FDK reconstruction, and the cone-beam backprojection steps of these two calculations can be combined to reduce the computation cost. Since in FBP reconstructions, the backprojection step takes the majority of the computation time, the computation complexity of the proposed reconstruction (24) is close to that of the FDK reconstruction only.

## 3. NUMERICAL RESULTS

### 3.1. Simulation details

The algorithm performance was evaluated using computer simulations. Table 2 summarizes the system parameters used in the simulations. Three computer phantoms were used in this study. The first was the 3D Shepp-Logan phantom as defined in [23], which contains low-contrast objects. The second was the FORBILD head phantom (http://www.imp.uni-erlangen.de/forbild/). This phantom contains high-contrast objects, and therefore it results in more missing data in circular CBCT geometry. To further verify the algorithm, the Defrise disc phantom was also used. The Defrise phantom consists of seven ellipsoidal discs stacked in the *z* direction. Each disc has a uniform attenuation coefficient of 1 mm^−1^, and the ellipsoid has a diameter of 140 mm and a thickness of 14 mm, with a distance of 25 mm between discs. This phantom has strong high-frequency components in the *z* direction, and therefore has high values in its first derivative of the Radon transform in the region where the data are unmeasured in a circular CB scan. It represents the most challenging case of reconstruction using the circular CB data.

Simulations were carried out on full-scan data. To test the stability of the algorithm, reconstructions on noisy data of the Shepp-Logan phantom were also investigated. In the simulation, we used 300 000 photons per ray, and the base material of the Shepp-Logan phantom is modeled as water at 80 keV, with a linear attenuation coefficient of 0.01837 mm^−1^.

To demonstrate the merit of the proposed algorithm, we compared the reconstructions using five different algorithms: the FDK algorithm [2], Hu's algorithm (only the first two terms of (24) are included) [5], the T-FDK algorithm [10], Yang's algorithm [19], and the proposed algorithm (24). All the five algorithms are in the category of analytical reconstruction algorithms for circular CBCT. As discussed earlier in the introduction section, the T-FDK algorithm was developed heuristically with a structure of shift-invariant FBP, and Yang's algorithm was based on interpolation in the Radon space, with a structure of shift-variant FBP.

### 3.2. Reconstruction results

**Figure 6:**
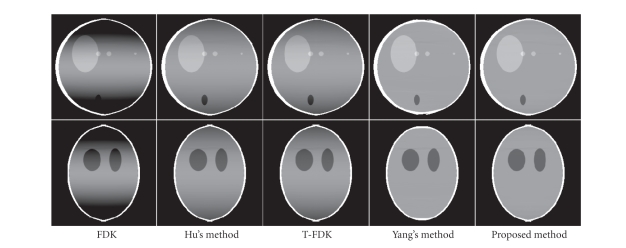
Reconstructions of the Shepp-Logan phantom. Top row: *x*-*z* views; bottom row: *y*-*z* views. Display window: [0.98 1.05].

**Figure 8:**
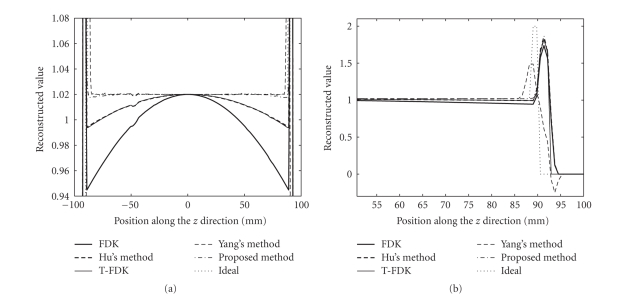
1D central vertical profile comparison of Figure 6. (a) The reduction of the axial intensity drop. (b) The reconstructed object edge using different algorithms.

**Figure 9:**
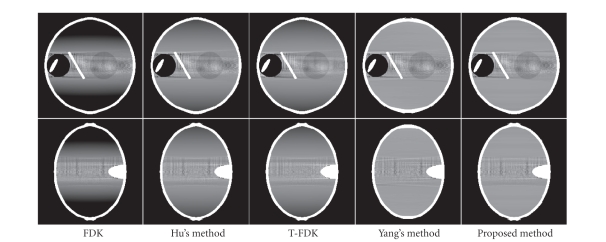
Reconstructions of the FORBILD head phantom. Top row: *x*-*z* views; bottom row: *y*-*z* views. Display window: [1.02 1.08].

**Figure 10:**
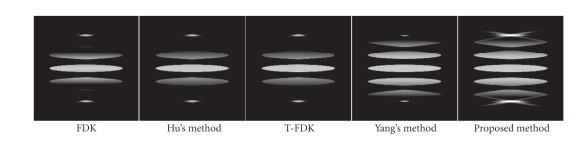
Reconstructions of the Defrise phantom, *x*-*z* views. Display window: [0.7 1.1].

**Figure 11:**
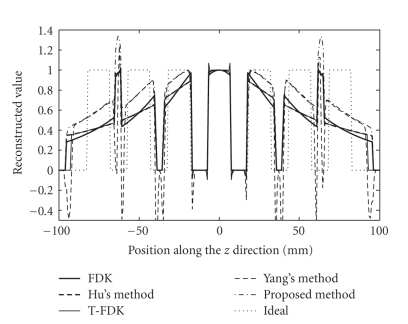
1D central vertical profile comparison of Figure 10.

Figures 6, 9, and 10 show the reconstructed images on a full scan of the Shepp-Logan phantom, the FORBILD head phantom and the Defrise disc phantom, respectively. Comparisons of 1D vertical profiles of these images are also shown in Figures 8 and 11. The algorithm performances on the FORBILD head phantom are close to those on the Shepp-Logan phantom, and the 1D profile comparison of the reconstructions of the FORBILD head phantom is omitted here.

From the comparison, it is seen that the CB artifacts of axial intensity drop are apparent in the reconstructions using the FDK algorithm or Hu's algorithm, and these artifacts are effectively suppressed using the proposed method.

The performance of the T-FDK algorithm on the reduction of intensity drop artifacts is also inferior to that of the proposed algorithm, as shown in the comparison of reconstructions. It is worth mentioning that the T-FDK algorithm is slightly more efficient than the FDK algorithm [10], although it causes resolution loss [18]. Recall that the proposed algorithm requires computation close to that of the FDK algorithm. Therefore, the T-FDK algorithm is more efficient than the proposed algorithm. Note that the reconstruction results using Hu's algorithm and the T-FDK algorithm are very similar. This similarity holds under certain conditions, and readers can refer to [24] for detailed discussion.

Compared to Yang's algorithm, our proposed algorithm has an advantage of high computation efficiency. As discussed in Section 2.4, the correction term in our algorithm only involves integration and derivative operations, and it is computed very efficiently. Yang's correction term has a structure of shift-variant filtering and backprojection; both steps require intense computation. In our implementations, Yang's algorithm is typically 7-8 times slower than our proposed algorithm.

Both the proposed algorithm and Yang's algorithm achieve similar reduction of the intensity drop away from the object edge. Nonetheless, estimation of high-frequency data in Yang's algorithm causes relatively large errors. The resulting artifacts are around the object edges in the axial direction, typically streaks with negative magnitudes. This problem can be seen in Figure 8(b), and is more obvious in Figure 11, where data estimation is more challenging due to the high-frequency missing data along the axial direction. Our algorithm does not estimate the high-frequency missing data, and the negative streaks do not appear around the object edges.

**Figure 12:**
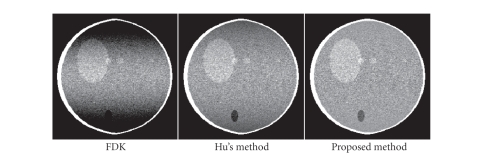
Reconstructions of the Shepp-Logan phantom, using noisy projection data, *x*-*z* views. Display window: [0.98 1.05]. Based on the noise-free reconstructions as shown in Figure 6, from left to right, the noise variances in the images are measured as 5.872 × 10^−5^, 5.872 × 10^−5^, and 5.874 × 10^−5^.

Reconstructions on noisy projection data are shown in Figure 12 to demonstrate the stability of the proposed algorithm in the presence of noise. The algorithm performance is similar to that in previous comparisons. Based on the noise-free reconstructions as shown in Figure 6, the noise variances in the images are also measured. The noise in the image using the proposed algorithm remains in the same level as that in the image using the FDK algorithm or Hu's algorithm.

## 4. CONCLUSIONS AND DISCUSSION

In this work, we propose an efficient estimation method to reduce the intensity drop in the CB reconstruction on circular scans. The algorithms are derived using data analysis in the Radon domain via Grangeat's formula. Assuming the CB projections are measured in a parallel beam geometry, we estimate the unmeasured data from the CB projections. These data are then reconstructed to form a correction term to improve the FDK reconstruction with Hu's term included. Equivalently, an implicit data interpolation/extrapolation is carried out in the Radon domain. It is interesting to note that Hu's term takes the first derivative of the projection data along the axial direction, while our correction term takes the second derivative. Although our algorithm is derived for circular CBCT on a full scan, it can be easily extended to short-scan reconstructions using weighting schemes, such as Parker's weighting.

The algorithm performances are evaluated using computer simulations on the 3D Shepp-Logan phantom, the FORBILD head phantom, and the Defrise disc phantom. The results show that the proposed method greatly suppresses the axial intensity drop in the FDK reconstructions and its performance improves on Hu's algorithm. Residual artifacts are mainly due to the high-frequency Radon space data missing in a circular CB geometry, which cannot be estimated accurately using interpolation or extrapolation in general. As demonstrated in the results of the Defrise disc phantom, relatively large reconstruction errors are expected around high intensity objects, such as bones in a clinical case, in the longitudinal direction.

Our algorithm also outperforms the T-FDK algorithm on the reduction of intensity drop artifacts. As compared to other existing algorithms, such as Yang's algorithm, that are based on interpolation in the Radon space, our algorithm has an advantage of high efficiency. The calculation of the correction term requires only simple integration, 1D derivative and multiplication operations, and the total computation of the proposed algorithm is close to that of the FDK algorithm. In our implementations, the proposed algorithm is 7-8 times faster than Yang's algorithm.

## Figures and Tables

**Figure 7:**
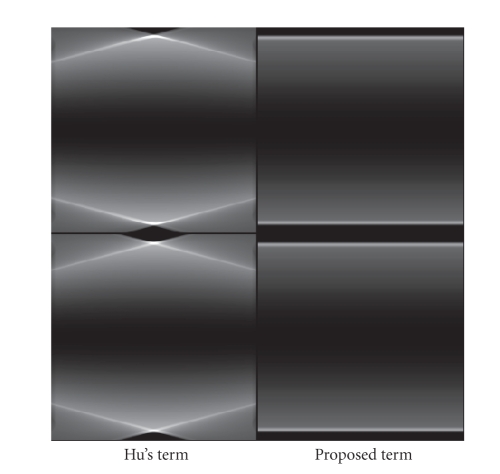
Images of the second term (Hu's term) and the third term (the proposed correction term) of (24), using the projection data on the Shepp-Logan phantom. Top row: *x*-*z* views; bottom row: *y*-*z* views. Display window: [0 0.08].

**Table 1:** Variable glossary.

*D*	Distance from the X-ray source to the rotation center
*D*_*od*_	Distance from the rotation center to the detector
*f*	Scanned object
${\hat{f}}_{c}$	Estimation of ${\hat{f}}_{N}$
f^FDK	Reconstruction using the FDK algorithm
${\hat{f}}_{H}$	Correction term in the Hu-FDK algorithm
${\hat{f}}_{N}$	Missing information in the circular CB trajectory
*g*_0_	Ramp filtering kernel
*g*_*h*_	Hilbert transform kernel
*p*	CB projection image
*p*_*F*_	Ramp-filtered image of *p*_*m*_
*p*_*h*_	Intermediate function defined in (17)
*p*_*m*_	Projection image reconstructed from partial data of *S*′ *p*_*p*_
*p*_*p*_	Parallel-beam projection image
*p*_*v*_	Projection image on the virtual detector (see Figure 1)
*R* *f*	Radon transform of an object *f*
*S* *p*	Weighted sinogram of a projection image *p*
*w*	Map function defined in (7)
*α*	Angular parameter of *S* *p*
*β*	CB projection angle
*γ*_*m*_	Full fan angle
*ρ*	Displacement parameter of *S* *p*

**Table 2:** Simulation parameters.

Source to detector distance (*D* + *D*_*od*_)	700 mm
Source to axis distance (*D*)	350 mm
Detector size	512 × 512
Detector element pitch	0.781 mm
Full cone angle in the *z* direction	32*deg*
Full fan angle in the *x*-*y* plane	32*deg*
Projection number of a full scan	800
Reconstructed volume	256 × 256 × 256
Reconstructed voxel size	0.781 mm
